# Waste management CDM projects barriers NVivo 10® qualitative dataset

**DOI:** 10.1016/j.dib.2017.10.014

**Published:** 2017-10-10

**Authors:** André Luiz Bufoni, Aracéli Cristina de Sousa Ferreira, Luciano Basto Oliveira

**Affiliations:** a Laboratory of Accounting Systems Modeling - Federal University of Rio de Janeiro, Brazil; b International Virtual Institute of Climate Change (IVIG), Brazil

## Abstract

This article contains one NVivo 10® file with the complete 432 projects design documents (PDD) of seven waste management sector industries registered as Clean Development Mechanism (CDM) under United Nations Framework Convention on Climate Change (UNFCCC) Kyoto Protocol Initiative from 2004 to 2014. All data analyses and sample statistics made during the research remain in the file. We coded PDDs in 890 fragments of text, classified in five categories of barriers (nodes): technological, financial, human resources, regulatory, socio-political. The data supports the findings of author thesis [1] and other two indexed publication in Waste Management Journal: “The financial attractiveness assessment of large waste management projects registered as clean development mechanism” and “The declared barriers of the large developing countries waste management projects: The STAR model” [2], [3]. The data allows any computer assisted qualitative content analysis (CAQCA) on the sector and it is available at Mendeley [4]

**Specifications Table**

**Table:** 

Subject area	*Environmental Sciences*
More specific subject area	*Waste Management*
Type of data	*NVivo® Qualitative Dataset*
How data was acquired	*UNFCCC CDM Project Search*
Data format	*Raw, Filtered and Coded (classified)*
Experimental factors	*432 Large Projects Design Documents (PDD) of Waste handling and disposal (13) Sector*
Experimental features	*The data was treated following the protocol: (1) the projects were imported to NVivo 10®, and (2) classified into seven categories according to the technology, (3) search for the term “barrier” in every document (1k+ fragments), (4) out of scope text fragments selected was discarded, (5) filtered data set (890 fragments) was coded into five barriers categories indicated in literature: technological, financial, human resources, regulatory, and socio-political.*
Data source location	*Global, Not applicable*
Data accessibility	*Bufoni, André (2017), “Waste Management Clean Development Mechanism CAQCA NVivo 10(R) Dataset”, Mendeley Data, v1*
	http://dx.doi.org/10.17632/n4jn2wfvg5.1

**Value of the data**

• The UNFCCC raw data source is mixed up, disconnected, fragmented, and laborious.
• This data is all inclusive (raw, coding, analysis and the research protocol is in one single file). Therefore, any other CAQCA can be immediately made for the WM sector.
• The data is standardized by UNFCCC, which means that it can be easily appended, replaced, compared or transposed to the others 14 sectors and 4.000 projects (external validity).
• The dataset is an instructive example of a Computer Assisted Qualitative Content Analysis (CAQCA) using the features of a CAQCA Software

## 1 Data

Despite a clear indication that waste management research needs a more system-oriented approach [5], environmental and technical aspects of the projects are usually considered in some case studies, or in a hermetic, static and disjointed way [6], [7]. Furthermore, databases and inventories, which can significantly aid policy makers in DC's are also scarce [2], [8], [9]. Thus, the UNFCCC database on Waste Management Clean Development Mechanism Projects is a comprehensive and significant information source for project designers, industries, policy makers and countries. However, the information is not tabulated, and simply a set of projects’ individuals, what makes any analysis time-consuming.

This data article shares, in a single NVivo 10® software file, 432 large waste management projects design documents (PDD) from 2004 to 2014 [4]. Previous research used this dataset to proceed a computer assisted qualitative content analysis (CAQCA) for assessment of projects financial attractivity [2] and to study the projects implementation barriers [1], [3], but the data supports any desired content analysis within the sector scope.

The sample selected from CDM Project Search site of UNFCCC [10] contains:

1) the entire PDD documents;
2) a qualitative data analysis (QDA) aiming the study of the project's designers declared barriers;
3) results of a primary search for the stated barriers (890 text fragments);
4) we code (hermeneutic) fragments into five barriers categories: technological, financial, human resources, regulatory, and socio-political.

The data is standardized following the main UNFCCC guidelines for any project [11], [12], [13], [14], which means that it can be easily appended, replaced, compared or transposed to the others 14 sectors and 4.000 CDM projects. It has a strong external validity regarding the CDM context.

## 2 Experimental design, materials and methods

The computer assisted qualitative content analysis (CAQCA) drives the data file design. CAQCA is a well-known methodology largely used in social applied and human sciences [15], [16], [17], [18]. The method involves the recursive procedure (coding) of a simple term search on the content to select the units for analysis (phenomenology) and, for those units, a semantic search, interpretation, and possible relationships among the fragments selected (hermeneutic) [19]. Bowen [20] highlights that the content analysis documents are also a source of a historical view, new hypotheses, modeling support, change and development tracking, and evidence triangulation.

The use of software (qualitative data analysis software - QDAS) to treat a large volume of data produced almost in real time had a significant impact on research institutions and doctoral programs because part of those research without the QDAS would be impractical and impossible. This fact explains why the more Universities offers the QDAS at an institutional level [21]. Furthermore, the development of specific software for content analysis made several searches and coding tools, statistics for hypothesis testing and concepts development available, that, in turn, have a significant impact on the research results [16], [22].

**Fig. 1:**
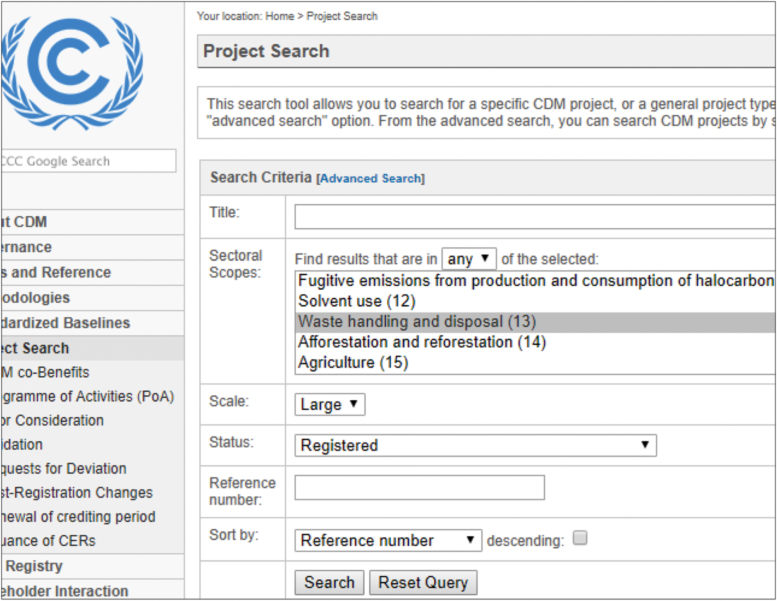
Projects Search Criteria. Source: UNFCCC [10]

The search criteria used to the content selection (raw data) from the UNFCCC Project Search site [10] in this file is in Fig. 1. Sectoral Scope: Waste handling and disposal (13), Scale: Large, Status: Registered, Sort by: Reference number. We choose the barriers from the literature and the official bodies' reports. The original studies supported by the data aimed the waste management sector barriers, but we can extend it to any other waste management projects aspect. Only large projects (432 of 923) were selected because only large projects must full disclosure technical and financial information about the initiatives, what makes the sample more useful.

The projects have many possible statuses (requesting registrations, deregistered, withdraw, rejected, revision, applied for, and corrections). We choose the registered status because no other guarantees UNFCCC consent for the registered operation. At last, the sort by reference number is strategic considering that the computer database key-index system is more reliable than registration date.

The results by the host country and year are in the original article [23]. Most projects are from China, Brazil, and Mexico (215). Beware of the year of the projects. Most of the Chinese projects are complex incinerators and took far more time to be available than other countries more straightforward and CAPEX lower projects. All incinerators are from China.

There is also an abnormal concentration of 124 projects in 2012 [1], possibly because it was the last year of the Kyoto Protocol first cycle (CP1). There was a relative uncertainty about the program continuity, which causes the investors run to apply before it is over [24].

After the simple search for the term ‘barrier’ that returned more than a thousand occurrences, took place the coding second part by semantic-hermeneutic analysis of fragments. Some of these fragments proof meaningless and discarded. The rest classified in categories for future interpretation and pattern identification. Table 1 summarizes the results.

**Table 1:** Fragment selected for analysis.

**Industry**	**Proj**	**PDDs**	**Barriers**				
			**Financial**	**Human Resources**	**Regulatory**	**Technological**	**Total**
Docs	432	254	88	78	71	137	890
Landfill	246	99	36	135	6	99	276
Animal Waste	60	55	3	17	79	30	129
Sewage	55	47	77	71	50	137	335
Composting	23	17	12	9	3	47	71
RDF	5	4	4	0	0	4	8
Incinerators	41	31	22	32	0	17	71
Gasification	1	1	0	0	0	0	0
**Total**	432	254	154	264	138	334	890
